# Fatal Spotted Fever Rickettsiosis, Minas Gerais, Brazil

**DOI:** 10.3201/eid0911.030193

**Published:** 2003-11

**Authors:** Márcio Antonio Moreira Galvão, J. Stephen Dumler, Cláudio Lísias Mafra, Simone Berger Calic, Chequer Buffe Chamone, Gracco Cesarino Filho, Juan Pablo Olano, David H. Walker

**Affiliations:** * Universidade Federal de Ouro Preto, Minas Gerais, Brazil; † World Health Organization Collaborating Center for Tropical Diseases, University of Texas Medical Branch, Galveston, Texas, USA; ‡ Johns Hopkins University, Baltimore, Maryland, USA; § Fundação Ezequiel Dias, Belo Horizonte, Minas Gerais, Brazil; ¶ Diretoria Regional de Saúde, Coronel Fabriciano. Minas Gerais, Brazil

## Abstract

The emergence and reemergence of a serious infectious disease are often associated with a high case-fatality rate because of misdiagnosis and inappropriate or delayed treatment. The current reemergence of spotted fever rickettsiosis caused by *Rickettsia rickettsii* in Brazil has resulted in a high proportion of fatal cases. We describe two familial clusters of Brazilian spotted fever in the state of Minas Gerais, involving six children 9 months to 15 years of age; five died. Immunohistochemical investigation of tissues obtained at necropsy of a child in each location, Novo Cruzeiro and Coronel Fabriciano municipalities, established the diagnosis by demonstration of disseminated endothelial infection with spotted fever group rickettsiae. The diagnosis in the two fatal cases from Coronel Fabriciano and the surviving patient from Novo Cruzeiro was further supported by immunofluorescence serologic tests.

Infection with *Rickettsia rickettsii,* known as Brazilian spotted fever (BSF) or Rocky Mountain spotted fever (RMSF), occurs in the United States, Canada, Mexico, Costa Rica, Panama, Colombia, Brazil, and Argentina (*1*–*3*). Investigations of RMSF often uncover several fatalities (*4*). A high case-fatality rate is associated with the emergence or reemergence of RMSF after a decade or more of low incidence. Subsequently, the public health concern and educational efforts usually lead to more effective diagnosis, antirickettsial treatment, and a lower case-fatality rate. BSF cases and outbreaks were described in Minas Gerais state, Brazil, beginning in 1929 and continuing until 1944. Then, and until 1980, no cases were described in the medical literature. Interviews with physicians in practice during this period disclosed only rare cases of BSF. Outbreaks occurred again in Minas Gerais state in 1981, 1984, 1992, 1995, and 2000. Although several cases in these outbreaks were fatal, the diagnoses were not well documented by laboratory methods except during the 1995 and 2000 outbreaks. The true mortality rate of RMSF is often hidden because autopsies are performed in only a low proportion of deaths (*5*). Immunohistochemical detection of *R. rickettsii* offers an accurate diagnosis both retrospectively in fatal cases and in cutaneous biopsies of lesions during acute illness (*6*). We describe two outbreaks of BSF in families that occurred in 1995 and 2000 in Novo Cruzeiro and Coronel Fabriciano municipalities of Minas Gerais state, Brazil; six pediatric cases, five fatal, were involved.

## Materials and Methods

**Figure 1:**
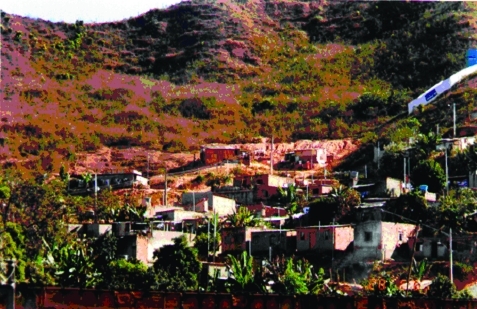
A characteristic habitat in spotted fever–endemic area, Brazil.

Novo Cruzeiro Municipality, located in one of the poorest areas of Minas Gerais state in the northeastern region, has a population of 35,000, who live mainly in rural areas. Coronel Fabriciano Municipality is located in Rio Doce Valley in the eastern part of Minas Gerais state. This region was industrialized 30 years ago, but transition areas with rural characteristics persist in the peripheral area of its cities. Horses are prevalent, a fact that plays an important role in supporting the *Amblyomma cajennense* tick population (Figure 1).

### Novo Cruzeiro Municipality

These cases all occurred in the same family in 1995, involving four boys of 9 months, 3 years, 4 years, and 5 years of age. All the patients died, except the 3-year-old boy; a sample of serum collected on day 5 of this boy’s illness was tested by indirect immunofluorescence assay (IFA) for antibodies to *R*. *rickettsii*. A necropsy was performed in the index patient, the 9-month-old boy, and tissue samples of skin, brain, stomach, liver, spleen, and kidney were collected. These materials were fixed in neutral-buffered formaldehyde and shipped to the Department of Pathology, University of Maryland School of Medicine, Baltimore, Maryland. Immunohistochemical examination for spotted fever rickettsiae was performed according to the method of Dumler et al. (*7*).

### Coronel Fabriciano Municipality

Twenty-one suspected cases of BSF in patients with fever and rash were reported in Coronel Fabriciano Municipality during 2000. Thirteen patients had a history of recent tick bite and came from Pedreira, a periurban area with rural characteristics. Among these 21 suspected patients, 2 children (12 and 15 years of age) died. They were brothers who lived in the same house. Fleas, collected from dogs in this house during the outbreak, had *R. felis,* as detected by polymerase chain reaction (PCR) (*8*). IFAs to detect antibodies to *R. rickettsii, R. typhi*, and *Ehrlichia chaffeensis* were performed on serum from all 21 patients (*9*). A second serum sample was obtained from nine patients. The reactive serum samples were also tested for antibodies to *R. felis*. A necropsy was performed on the second fatal patient, and samples of skin, brain, stomach, liver, spleen, and kidney were collected. These materials were fixed in neutral-buffered formaldehyde and shipped to the Rickettsial and Ehrlichial Diseases Research Laboratory, Department of Pathology, University of Texas Medical Branch at Galveston, Texas. Immunohistochemical examination for spotted fever rickettsiae was performed by using a monoclonal antibody against a lipopolysaccharide epitope distinctive for spotted fever group rickettsiae (*10*). PCR was attempted to amplify rickettsial DNA from formalin-fixed, paraffin-embedded necropsy tissues from this patient (*11*).

## Results

**Figure 2:**
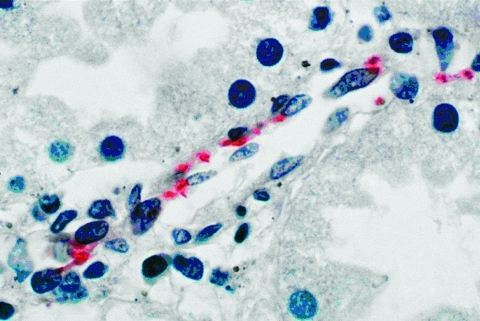
Immunohistochemical stain demonstrates *Rickettsia rickettsii* in endothelial cells of a blood vessel in kidney from patient l. (Hematoxylin counterstain; original magnification X 1200).

In Novo Cruzeiro Municipality, the index patient, a 9-month-old boy, was seen with fever and cough of 5 days’ duration; a rash had developed on day 3 of illness. On physical examination, a high fever, maculopapular exanthem, diarrhea, and coma were noted. Four days later, the patient was in hypotensive shock, had a seizure, and died. The other three brothers had fever, nausea, vomiting, and a maculopapular rash. All the brothers had a history of tick bite in the 15 days before the onset of symptoms. The 4- and 5-year-old children died in hypotensive shock on days 8 and 9, respectively, after onset of disease. An IFA antibody titer of 512 against *R. rickettsii* developed in the surviving 3-year-old child 5 days after the onset. A sample of serum collected 6 months later from this patient showed an IFA antibody titer of 8,192 against *R. rickettsii*. Vascular endothelial cells in the liver, stomach, and kidney of the index patient contained spotted fever group rickettsiae demonstrated by immunohistochemical results in multiple foci of lymphohistiocytic vasculitis (Figure 2).

In Coronel Fabriciano Municipality, the first person to become ill was a 12-year-old boy; during the course of disease he had fever, nausea, vomiting, diarrhea, abdominal pain, headache, myalgias, and edema. Before death, stupor and renal failure occurred. Subsequently, fever, rash, nausea, vomiting, diarrhea, abdominal pain, headache, myalgia, jaundice, and renal failure occurred in the 15-year-old brother of the index patient, and he also died. Both patients reported a tick bite. Immunohistochemical evaluation of the necropsy materials obtained in the second fatal case, using an immunoglobulin (Ig) M monoclonal antibody against a lipopolysaccharide epitope specific for *Rickettsia* of the spotted fever group, demonstrated typical rickettsiae in vascular endothelium. Attempted PCR failed to amplify rickettsial DNA from tissues in the case from Coronel Fabriciano, presumably owing to the quality of DNA in the formaldehyde-fixed, paraffin-embedded blocks.

Among 21 suspected cases of spotted fever rickettsiosis in the second half of 2000 in Coronel Fabriciano, serum samples from three patients contained antibodies to *R. rickettsii* detected by IFA in the first sample at a titer of 64, including the serum of one patient who died; 13 of these patients reported tick bites. A second sample of serum was collected for testing for antibodies to *R. rickettsii* in nine of these cases. The second serum sample of one patient, whose first sample was negative, reacted at a titer of 64 on day 12 of disease. Among the three patients whose first samples contained antirickettsial antibodies, a second sample was not collected in two cases, and in one case the titer did not increase when the sample was tested 12 days later. The three patients with IFA antibodies to *R. rickettsii* had fever, headache, and rash. None had IFA antibodies to *R. typhi* or *E. chaffeensis***.** The serum samples that reacted with *R. rickettsii* did not contain antibodies to *R. felis* detected by IFA.

## Discussion

*R. rickettsii* causes the spotted fever rickettsiosis with the highest case-fatality rate. In Brazil, *R. felis* is the only other spotted fever group rickettsia documented to cause human disease, whereas in the United States rickettsialpox also causes rickettsiosis (*1*,*12*). Although these diseases are not distinguished by IFA serologic tests, unless cumbersome absorption studies are performed with specific antigens, these five children undoubtedly died of fatal *R. rickettsii* infection.

BSF has been reported in the Brazilian states of Minas Gerais, São Paulo, Rio de Janeiro, Espirito Santo, and Bahia (*13*), where it is transmitted by *A. cajennense* ticks (*14*). These ticks are distributed from northern Argentina to southern Texas and could harbor *R. rickettsii* at any location in between. In the United States, *R. rickettsii* is maintained transovarially and transtadially in *Dermacentor* ticks such as *D. andersoni* and *D. variabilis*, although maintenance by means of rickettsemic mammals may also play a major role (*15*). Only a small fraction, most likely <0.1%, of *A. cajennense* and *D. variabilis* ticks carry *R. rickettsii,* which affects ticks as well as humans (*16*–*18*). Thus, investigations of tick populations even in the vicinity of cases of RMSF or BSF do not necessarily detect ticks infected with *R. rickettsii.*

The phenomenon of familial clusters of RMSF has been noted numerous times. In fact, the simultaneous occurrence of severe febrile illness in more than one patient generally suggests person-to-person or a point-source transmission of infection. Few physicians may be aware that 4.4% of cases of RMSF occur in the household of another case-patient with the disease (*15*), a situation that often lends further diagnostic confusion for this illness that can mimic other febrile exanthems, such as dengue, as well as gastrointestinal infection, other abdominal conditions, pneumonia, and meningoencephalitis (*19*–*22*). We realize that some patients with suspected BSF from Coronel Fabriciano Municipality, Brazil, with fever and rash and without seroconversion by IFA to *R. rickettsii,* might have another disease. Dengue fever is also endemic in this region in some periods of the year.

In the United States, the incidence of RMSF undergoes cyclic periods of increase and subsequent decrease extending over decades (*23*). The consistently rising incidence of RMSF in the United States in 1999 and 2001 suggests that reemergence is occurring. Likewise, these outbreaks of fatal cases in children from Minas Gerais state, Brazil, may indicate a reemergence of BSF in that country. Although we do not have an incidence rate for BSF documented in the 1980s, BSF surveillance was implemented during that period. As a result, an incidence rate of 0.35 BSF cases per 100,000 population has been estimated for the early 1990s (*13*). A high case-fatality rate of 40% for BSF in Minas Gerais state between 1981 and 1989 also suggests reemergence of this disease.

The mechanisms underlying reemergence and subsidence are not known, but several factors, including suburbanization, destruction of the forests, and increased outdoor activities appear unlikely to be involved. None of these factors decreased markedly during the 1980s and early 1990s, when the incidence of RMSF waned. Now may be an appropriate time to investigate the ecology of *R. rickettsii* as well as to mount a campaign of increased public and physician education regarding RMSF and BSF to avoid deaths from delayed or missed diagnosis of this disease, which is usually difficult to diagnose in its early course. Emphasis should be placed on initiation of therapy with doxycycline in the first 4 days of illness, which dramatically reduces the case-fatality rate of this disease.
